# Notes and News

**Published:** 1892-03-26

**Authors:** 


### Notes and News.

Collected and Communicated.

A bill is to be brought forward by Mr. Mather, M.P., in which it is proposed to confer upon the more important local authorities—namely, county councils, borough councils, and urban sanitary authorities—the power of contributing to the support of those hospitals, infirmaries, &c., which are maintained by voluntary subscriptions, and are not carried on for private profit. The terms upon which a subscription may thus be made are to include one that the local authority shall be represented on the governing body of the institution in such proportion as may be agreed on.

Lord Calthorpe announces that it has been decided to establish a memorial fund, the proceeds of which shall be devoted to some permanent addition to the Throat Hospital, Golden Square, in memory of Sir Morell Mackenzie, an institution which he founded, and to which for a period of twenty-seven years he devoted the greater part of his time, the best of his energies, and the whole of his heart. The memorial fund will be kept entirely separate from the hospital funds, and will be permanently identified with Sir Morell Mackenzie's name.

The jubilee extension of the Aberdeen Infirmary is almost completed, and the new buildings are expected to be opened for general use in the month of May. There is one part of the building already completed and partly in use—the pathological and laundry block. This block consists of the mortuary and funeral service-room; pathological store; post-mortem theatre; laboratory; professors' suite of rooms; laundry accommodation; and disinfecting rooms. For disinfecting purposes there are two rooms set apart. The disinfecting apparatus is a Washington Lyon's patent steam disinfector. The apparatus consists of a steam jacket and a chamber containing a cage, into which the articles are put that are to be disinfected, and has two doors—one opening into the room where the infected articles are kept, and the other connected with the second apartment, thus allowing of the articles being taken out into a fresh room. Steam is admitted to the jacket, or casing surrounding the chamber, until the desired temperature is attained. When this is reached, steam is admitted to the inner chamber itself, and so comes in direct contact with the articles to be dealt with. The process lasts over fifteen minutes, and the steam is then let off. The steam remains for a short time longer in the outer chamber, thus forming a drying chamber. In the laundry the Decodin machine ironers are used, and Bradford's patent ironing stones and moveable linen airer. The pathological department is very complete and ample accommodation and the best appliances are provided.

The new buildings for the National Dental Hospital will be commenced immediately, and will consist of a basement and three upper floors, providing accommodation for seventy students; the buildings will be fitted with the electric light, white enamel bricks will be used throughout. The capacity of the present hospital has long been greatly overtaxed, and the new buildings are urgently needed. We are informed that the liberality of a lady who has long taken a great interest in the institution has relieved the Committee of any serious financial anxiety in connection with the movement. The efficiency of the hospital as a school of dentistry and the comfort of the patients will be greatly enhanced when the buildings are ready for occupation.

The annual meeting of the Governors of the New Hospital for Women, Euston Road, N.W., took place on Monday afternoon. The Rev. Llewellyn Davies was Chairman. The Dowager Lady Stanley of Alderley, Hon. Mrs. Pereara, Miss Emily Davies, Mrs. Garrett Anderson, Mrs. Westlake, Rev. A. B. Boyd-Carpenter, Mr. A. G. Pollock, and others attended. The annual report was presented, and was found to show a steady increase in the numbers of both the in-patients and out-patients treated in the hospital. This, of course, entails constantly greater expenses, and the hope was expressed by the Governors that this would be considered by subscribers and the public generally. Mr. Llewellyn Davies, who has been a warm supporter of this institution since its foundation, announced his satisfaction at the singular success of the whole enterprise.

At the last meeting of the Metropolitan Asylums Board an important subject was brought under notice through the report of the Ambulance Committee. It was stated that one effect of the passing of the Public Health Act, was to limit somewhat the scope of the powers of the managers for the conveyance of persons suffering from infectious diseases. It was now necessary, in order to extend the list of such diseases beyond those expressly named in the Act, that the London County Council should make a formal order to that effect. It seemed to the Committee a subject of regret that the Legislature were not prepared to take a bolder step in order to preserve from the taint of infection the public carriages of the metropolis. This is a serious matter, affecting as it does a large part of the community, and any act which renders evasion of proper precautions must be regarded in the light of a retrograde movement, which, it must be hoped, will be remedied at once.

The annual general meeting of the Royal London Ophthalmic Hospital was held last week. The report showed that the number of those suffering from diseases of the eye who had sought the aid of the hospital had been 27,450, including 1,892 in-patients. Operations to the number of 2,126 had been performed, giving sight again to many persons who had been deprived of it, and averting blindness from a large number. The medicine and drugs prescribed had been in every case provided free of cost to the patients, and 1,230 of those who were ordered spectacles had been either presented with them gratuitously or materially assisted in obtaining them. The number of artificial eyes given to patients was 102. The receipts for the year had been £4,681, as compared with £5,446 in 1890. The falling off of the income arose from a decrease in the amount of legacies, those for 1890 being £2,080, and for 1891 only £1,300. The annual subscriptions in 1891 amounted to £817, against £806 in 1890. Donations, which in 1890 were £1,687, increased in 1891 to £1,786. The sum required for re-building is £35,000.

